# The Impact of Real‐Time Visual Feedback on Maximal Force Output and Reliability During Isometric MidThigh Pull Testing in Resistance‐Trained Men

**DOI:** 10.1002/ejsc.70000

**Published:** 2025-07-01

**Authors:** Nicolay Stien, Atle Hole Saeterbakken, Vidar Andersen, Tom Erik Jorung Solstad

**Affiliations:** ^1^ Department of Sport, Food, and Natural Sciences Western Norway University of Applied Sciences Sogndal Norway

**Keywords:** measurement, strength, technology

## Abstract

Accurate and valid assessment of the maximal force production is essential for athlete monitoring and training prescription in sports science. This study investigated the impact of visual feedback on force output and measurement reliability in isometric mid‐thigh pull (IMTP). Twenty resistance‐trained men completed three variations of the IMTP test (single, repeated, and 30 s all‐out) across four sessions, with two sessions conducted with feedback and two without. Peak and mean force output was analyzed using peak and mean values. Data from the best testing day (i.e., highest force output in each variation) were used for comparisons between conditions, whereas test–retest reliability was assessed using data from the two sessions under the same condition. Visual feedback significantly enhanced most measures of peak and mean force outputs in all test variations (effect sizes ranging from 0.49 to 1.13 and *p* < 0.001–0.006). Reliability analyses of the single and repeated repetitions revealed that feedback reduced coefficients of variation (range: 2.57%–5.17% vs. 3.11%–6.92%) and yielded higher intraclass correlation coefficients (range: 0.961–0.983 vs. 0.898–0.987), indicating improved consistency both within sessions and between testing days. However, in the 30 s all‐out test, feedback did not significantly improve reliability, possibly due to pacing strategies influenced by the real‐time display. These findings demonstrate that real‐time visual feedback enhances both performance and reliability in strength testing, with important implications for research and applied sports science.

## Summary

- Real‐time visual feedback significantly increases peak and mean force output during isometric mid‐thigh pull testing.
- Visual feedback improves test–retest reliability in both single and repeated IMTP trials.
- No meaningful reliability improvements were observed during a 30 s all‐out IMTP despite higher force output with feedback.
- These findings support the use of visual feedback in performance assessments to enhance both force production and measurement consistency.

## 1 Introduction

Feedback is a key element in motor learning and performance enhancement, influencing attentional focus and movement execution (Pacholek and Zemková 2022; Randell et al. 2011; Sigrist et al. 2013; J. Weakley et al. 2023; Wulf et al. 2001). In strength testing and training, feedback can be provided in various forms, including verbal encouragement, visual cues, or real‐time feedback, from technological systems displaying performance metrics (J. Weakley et al. 2023, 2020). In isometric contractions, for example, displaying a live torque trace increased peak torque and rate of tension development (Hald and Bottjen 1987). In repeated high‐intensity squats, on‐screen barbell‐velocity feedback attenuated fatigue‐related velocity loss (J. J. S. Weakley et al. 2019), and in sustained isometric efforts, real‐time force cues supported accuracy under fatigue (Limonta et al. 2015). Beneficial effects of continuous feedback have also been observed in sprinting, jumping, and weightlifting (Concon et al. 2024; Hopper et al. 2003; Keller et al. 2014; Kellis and Baltzopoulos 1996; Pacholek 2024; Weinstock‐Zlotnick et al. 2011; Wilson et al. 2017). However, despite its potential benefits, the effect of real‐time visual feedback on performance and reliability in maximal isometric strength tests, such as the isometric mid‐thigh pull (IMTP), remains unclear.

Maximal strength is a central component of athletic performance, such as sprinting and jumping (Suchomel et al. 2016), and accurate assessment of maximal strength is essential for monitoring progress and predicting performance outcomes (Naclerio Ayllón et al. 2009). The IMTP has gained recognition as a reliable and valid method for assessing maximal isometric strength due to its strong associations with dynamic performance measures (Brady et al. 2020; Comfort et al. 2019; Drake et al. 2017; Grover et al. 2024; Pasfield et al. 2024; Verma et al. 2010). However, strength is not a singular construct, and different IMTP testing protocols may assess distinct aspects of an athlete's force‐generating capacity. For instance, single‐repetition IMTP primarily assesses maximal force production, whereas repeated IMTP trials and an all‐out IMTP effort measures muscular endurance (Grover et al. 2024). Interestingly, Pacholek (2024) found that real‐time quantitative feedback during bench press training drove greater strength and power gains than verbal or no feedback, whereas Randell et al. (2011) reported only modest improvements from peak‐velocity feedback in squat jumps and sprints. These outcomes may suggest that real‐time visual feedback does not produce uniform benefits across testing variations, highlighting the need to compare its effects on both performance and reliability in single‐ and repeated‐repetition tests, and all‐out tests.

Test–retest reliability ensures that observed changes in performance reflect true physiological adaptations rather than inconsistencies in testing conditions (Brady et al. 2020; Grgic et al. 2020). The reliability of assessing strength endurance may vary depending on the implementation of the test. Grover et al. (2024) found that a protocol consisting of repeated IMTP trials provided good reliability for strength endurance measures in repeated efforts (intraclass correlation coefficient [ICC] = 0.790), whereas a single 60 s prolonged IMTP effort showed poor reliability (ICC = 0.305). In other isometric tasks, Kim and Kramer (1997) demonstrated that visual feedback improved ICCs for knee‐extension torque across multiple sessions, indicating feedback can bolster consistency when baseline variability exists. In the less variable context of grip strength (ICCs ≥ 0.95), Ties Molenaar et al. (2010) found no further improvements in reliability from feedback, suggesting that its effects emerge with intrinsic variability. This underscores the importance of protocol selection based on the specific strength qualities and task complexity and highlights the need to investigate whether real‐time visual feedback can enhance reliability across IMTP protocols with differing baseline consistency.

The primary aim of this study was to examine the influence of real‐time visual feedback on maximal IMTP performance and measurement reliability. Real‐time visual feedback was provided via a live force curve to assess its influence on force output and measurement reliability in a single‐repetition IMTP, repeated IMTP trials, and a 30 s all‐out IMTP effort. Continuous visual feedback has been shown to enhance performance in a variety of tasks, including weightlifting and jumping (Concon et al. 2024; Weinstock‐Zlotnick et al. 2011; Wilson et al. 2017). In isometric testing, repeated‐effort protocols demonstrate good intersession reliability, whereas prolonged all‐out tests may suffer poor consistency (Grover et al. 2024). Given that feedback can both boost output and, by providing ongoing cues, potentially stabilize performance across trials, we hypothesized that real‐time visual feedback would increase peak and mean force production in single‐repetition, repeated‐effort, and 30 s all‐out IMTP protocols and improve both intrasession and intersession reliability of these measures. The findings from this study may help refine practices for isometric testing in research and applied performance settings.

## 2 Methods

### 2.1 Experimental Design

A repeated‐measures design was used to assess the influence of real‐time feedback on performance and reliability in an isometric test of maximal mid‐thigh pull strength. Before the experimental testing began, all participants were made familiar with the exercise and the feedback method. Each experimental session included a progressive warm‐up, and the testing was carried out in four sessions in a randomized and counterbalanced order, separated by 2‐to‐4 days of rest.

## 3 Participants

A convenience sampling strategy was employed to recruit participants from gyms near the university. A power analysis was conducted to determine the required sample size using a two‐tailed *t*‐test to detect differences between conditions. Given the previously reported effect sizes of 0.38 (Concon et al. 2024) and 0.87 (Weinstock‐Zlotnick et al. 2011) for visual feedback in strength‐related tasks, an average expected effect size of 0.625 was used as a conservative estimate. With a power of 0.80 and an alpha level of 0.05, calculations indicated that 14 participants were required to detect statistical differences between feedback conditions. A total of 20 resistance‐trained men volunteered to take part in the study (age: 28.4 ± 8.6 years, height: 180.0 ± 6.7 cm, body mass: 81.4 ± 9.4 kg, and resistance training experience: 9.9 ± 8.4 years). Participants were required to be familiar with resistance training and be free of any illnesses or injuries that could limit their maximal effort during testing. Prior to the commencement of the test, the participants were briefed both verbally and in writing about the study's protocols and objectives and they signed an informed consent form. The protocols conformed to the latest revision of the Helsinki declaration, followed Norwegian laws and regulations, and were processed by the Norwegian Center for Research Data (SIKT; reference: 140165).

### 3.1 Procedures

The first session included familiarization with the IMTP exercise and the real‐time feedback of the force output. After the test leaders had demonstrated two attempts, the familiarization for participants involved a gradual increase in the intensity of pulling on the bar while receiving instructions regarding positioning. Each participant's individual settings in the apparatus were also recorded during familiarization. Omitting a separate familiarization session was a deliberate choice to prevent potential confounding effects on performance or reliability due to prior experience with the testing procedures, so that the order of testing conditions (with vs. without feedback) would be the main difference between sessions. Previous investigations of the IMTP test support this as a time‐efficient approach that does not compromise reliability (Grgic et al. 2022). The testing procedures were identical between conditions, except that in two sessions, the participants were given visual feedback during the trials, whereas in the other two sessions, no feedback or information about their performance was given. To maintain consistency, verbal encouragement was withheld in both conditions.

The warm‐up for all sessions was identical, consisting of 10 dynamic deadlift repetitions at 50% of self‐reported one repetition maximum, followed by three 5‐s IMTPs at 50%, 70%, and 90% effort with 1 min rest between each pull (Dos’Santos et al. 2016). The order of the conditions (i.e., with or without feedback) was randomized and counterbalanced. Three different IMTP variations were tested in each session: 5 s maximum voluntary contraction (MVC), repeated MVC, and 30 s all‐out. Informed by previous anaerobic performance and fatigue studies, the repeated MVC test was designed to resemble established approaches to repeated sprint testing (Girard et al. 2011) and the 30 s MVC aligns with short‐duration all‐out tests used in cycling (Bar‐Or 1987), which has previously been successfully adapted to a continuous jumping test (Dal Pupo et al. 2014) to assess power output and force decline under prolonged maximal effort.

The single 5 s MVC was performed three times, with 90 s of rest between trials. Test leaders counted down the 3 s before initiating each MVC and the final 3 s of each effort. The 90 s rest period was deemed appropriate, as the brief MVC trials were unlikely to induce significant fatigue (Alexander et al. 2022). Furthermore, our data showed only a minimal decline in force across the three MVC trials, suggesting that 90 s of rest was sufficient in this context. The best attempt occurred first in 47.5% of trials, second in 32.5%, and third in 17.5%, with the average force output of the second and third attempts reaching 98.5% and 98.3% of the first attempt, respectively. Following 4 min of rest after the final single MVC, participants performed a repeated‐MVC bout comprising six 5 s MVCs separated by 30 s rest. A 30 s interval is widely used in repeated‐effort protocols and it yields higher peak and mean power and attenuates fatigue versus shorter recoveries in cycling (Glaister et al. 2005). In addition, the interval duration provokes greater metabolic stress and performance decrements than longer rest in heavy resistance training (Gerosa‐Neto et al. 2016). To ensure consistency, test leaders provided a 3 s countdown before each repetition and during the final 3 s of each pull. Following another 4 min rest, participants performed the 30 s all‐out MVC. The duration was chosen to mirror established short‐duration anaerobic protocols such as the Wingate test (Bar‐Or 1987) and their adaptations in jumping tasks (Dal Pupo et al. 2014). Throughout this effort, test leaders announced the remaining time at 5 s intervals and delivered a final 3 s countdown at the end of the trial to help participants sustain effort through completion.

**FIGURE 1:**
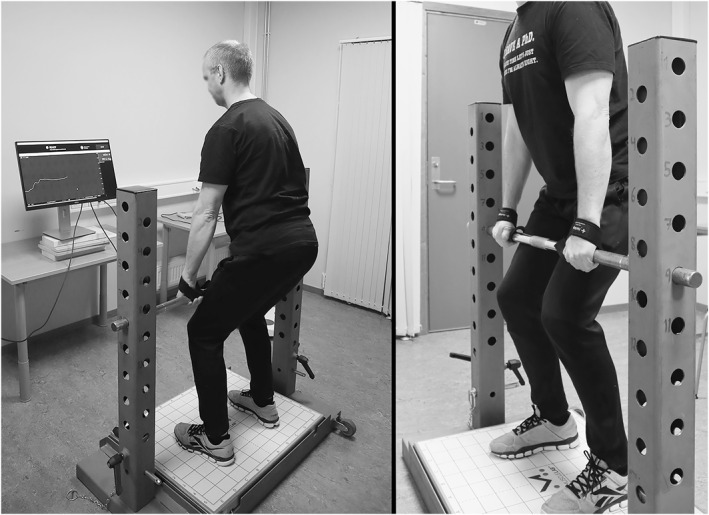
The position of the participants during the isometric mid‐thigh pull testing.

Participants used an overhand grip and wore lifting straps to eliminate grip fatigue as a limiting factor during testing (Comfort et al. 2019). The feet were placed directly below the bar (32 mm grip diameter), and the height of the bar was set approximately at the midpoint between the patella and the pubic bone (Figure 1). The degree of knee‐ and hip‐flexion was self‐selected but had to be within the recommended limits of 125°–145° and 140°–150°, respectively (Brady et al. 2020; Comfort et al. 2019). The joint angles, foot, and hand‐placements were measured during the first session and maintained in all subsequent trials. During the first session, a manual goniometer was used to measure each participant's joint angles and the exact bar height and knee/hip angles were recorded. Shoulder‐width hand spacing was measured at that time using a tape measure, and the measures were used to replicate these positions in all subsequent trials. Throughout testing, the experimenter visually monitored limb placements and joint angles immediately before each pull and continuously during each contraction to ensure participants maintained the prescribed positions and did not alter joint angles to gain mechanical advantage.

All trials started with the participants establishing a grip that they were satisfied with and confirming their readiness to start. The test leader then commenced the test by telling the participants to “push down in the force platform with the legs as hard as possible” when they were ready and maintain maximal effort until a signal to stop was given. To ensure standardized conditions for all participants, no verbal feedback, encouragement, or information about their performance was given during or after the attempts. However, the test leader verbally announced the remaining time of the effort.

Testing was conducted using a force platform (Ergotest Innovation A/S, Porsgrunn, Norway) with a 1000 Hz sampling frequency, positioned directly under an Olympic barbell. Force data were recorded using MuscleLab v. 10.4 (Ergotest Innovation A/S, Porsgrunn, Norway), and body mass was subtracted from all force measurements. The three IMTP tests were performed in a fixed order on four separate days, with feedback provided on two of those days. For condition comparisons, data from the testing day with the highest mean force output from each test variation was selected, whereas test–retest reliability was assessed by comparing data from both testing days within the same condition.

Peak force was defined as the highest recorded force during each trial, and the mean force was calculated as the average force output within the 3 s window of highest force output. To allow for a more stable comparison across trials, both the average peak and average mean forces were also calculated by taking the average collected from the three single MVCs. The same force parameters were extracted for each repetition in the repeated MVC trial, and analyses were conducted both at the repetition level and by averaging peak and mean force values across all six repetitions. For each condition in the single and repeated MVCs, the trial day yielding the highest force output was used in the feedback versus no feedback comparisons. During the 30 s all‐out MVC trial, the overall mean force was calculated over the central 25 s of the effort, excluding the initial and final 2.5 s to account for the force buildup at the start and the common end‐spurt near completion. To assess fluctuations in force output, the 3 s windows with the highest and lowest mean forces were identified. Additionally, the trial was divided into five consecutive 5 s intervals for the analyses, and mean force was calculated for each interval to examine the progression and decline of force over time. This structured approach ensured a comprehensive evaluation of force output and consistency across different testing conditions while distinguishing between real‐time visual feedback and its absence.

### 3.2 Feedback

Feedback was provided via a 24″ screen at chest height, positioned 1 m in front of participants at chest height, displaying both the live force‐time curve and numerical force output via the MuscleLab software. The vertical‐axis scale with labeled tick marks was visible at all times, and auto‐scaling ensured optimal zoom for real‐time viewing. During each 5‐s MVC, the full force curve was shown, and in the 30 s all‐out test, the display continually updated to show the most recent 10 s of data. Although participants could see the axis scale, they generally seemed to attend to curve amplitude and steadiness. Although no formal attentional control (e.g., eye‐tracking) was used, experimenters visually confirmed that participants were looking at the screen during feedback trials and debriefed them afterward to ensure they had monitored the force curve.

### 3.3 Statistical Analysis

All statistical analyses were conducted using SPSS version 27 (IBM Corp. Released 2020. IBM SPSS Statistics for Windows, Version 27.0. Armonk, NY: IBM Corp). Data distribution was assessed visually and using a Shapiro–Wilk test, confirming normality for all variables (*p* > 0.05). Relative reliability was assessed via ICCs for absolute agreement, and absolute reliability was quantified via coefficients of variation (CV). Both intersession reliability (between testing days) and intrasession reliability (across the three single‐MVCs and six repeated‐MVCs within the same day) were examined using these metrics. The ICC for absolute agreement was chosen because it accounts for both random and systematic errors, thereby providing a robust measure of reliability. The CV values were used to complement the ICC by expressing the degree of variability relative to the mean performance. Following the recommendations of a recent review of isometric strength testing (Brady et al. 2020), ICC above 0.8 and CV below 10% were considered indications of reliable measurements. Two‐factor repeated‐measures analyses of variance (ANOVA) (Condition × Session) were used to test the effects of visual feedback (feedback vs. no feedback) and session (Day 1 vs. Day 2) on each performance metric. Significant omnibus effects were followed by paired samples *t*‐tests as post hoc comparisons. Effect sizes for ANOVAs are reported as partial *η* ^2^. Effect sizes between conditions were calculated using Hedges' *g* by dividing the mean difference between conditions by the pooled standard deviation. The ES were interpreted with thresholds of < 0.2, 0.2–0.5, 0.5–0.8, and > 0.8, indicating trivial, small, medium, and large ES, respectively (Cohen 1998). Bonferroni corrections were applied to adjust for multiple comparisons and maintain control over the family‐wise error rate. Statistical significance was accepted at *p* < 0.05, and the Bonferroni‐adjusted *p*‐values are presented in the results.

## 4 Results

### 4.1 Force Output Comparisons

**FIGURE 2:**
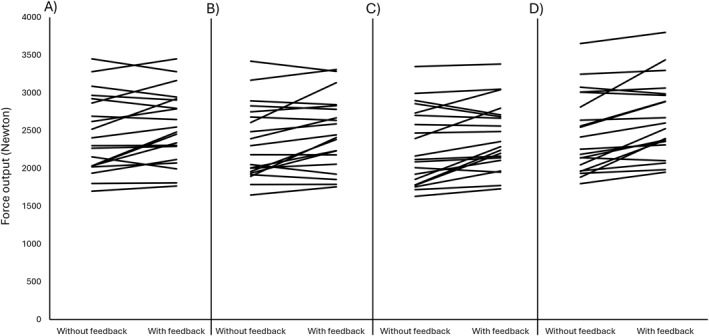
(A) The peak force output in the best attempt, (B) the average peak force output over three attempts, (C) the mean force output in the best attempt, and (D) the average mean force output over three attempts.

**FIGURE 3:**
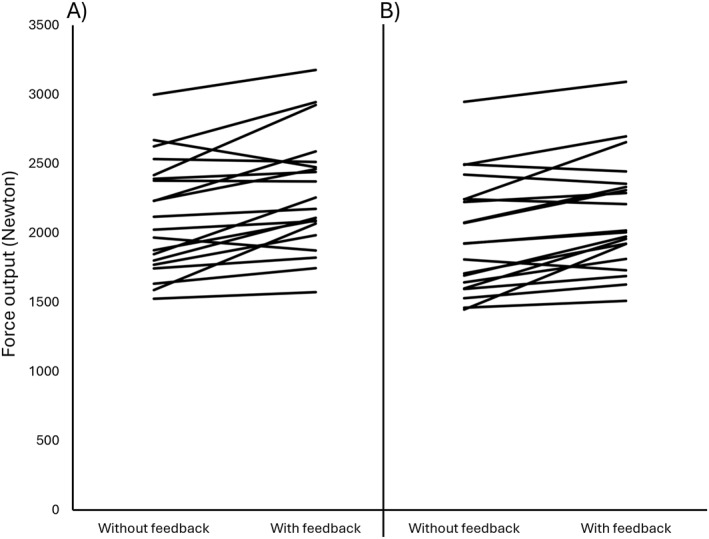
The average (A) peak and (B) mean force output (Newton) over the six repeated maximal voluntary contractions for the two conditions.

**FIGURE 4:**
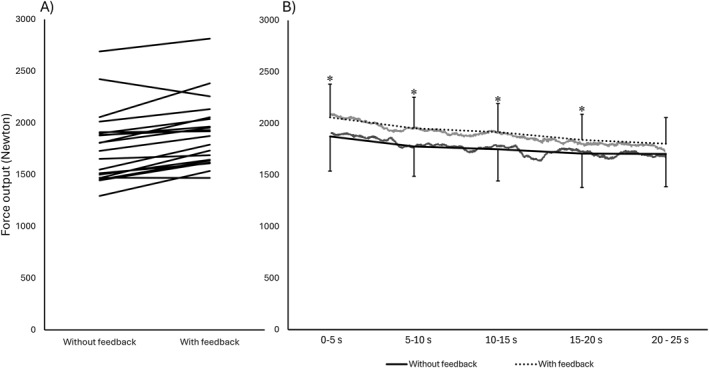
Individual changes between conditions in average force output (Newton) over the 25 s of effort (A) and the average force output within each of the 5‐s intervals (B) with raw data are overlaid in light gray (with feedback) and dark gray (without feedback). *Significant difference between conditions (*p* < 0.010).

An overview of the force data are presented in Table 1, and individual data are presented in Figures 2, 3, 4. The analyses of best peak force revealed a significant main effect of condition (*F*(1,19) = 10.23, *p* = 0.005, and partial *η* ^2^ = 0.35) but no main effect of session (*F*(1,19) = 0.01, *p* = 0.915, and partial *η* ^2^ < 0.01) nor a condition × session interaction (*F*(1,19) = 0.03, *p* = 0.874, and partial *η* ^2^ < 0.01). For mean force, a main effect of condition was found (*F*(1,19) = 10.07, *p* = 0.005, and partial *η* ^2^ = 0.35) but no effect of session (*F*(1,19) = 0.63, *p* = 0.438, and partial *η* ^2^ = 0.03) and no interaction (*F*(1,19) = 0.48, *p* = 0.496, and partial *η* ^2^ = 0.03). Post hoc tests confirmed that mean and peak force outputs were 4.7%–8.4% higher with feedback compared to no feedback (ES = 0.49–0.92 and *p* = 0.002–0.042; Figure 2).

**TABLE 1:** Comparisons of force output between the feedback and no‐feedback conditions presented as Newton ± standard deviation, with *p*‐values and effect sizes for the between‐conditions differences.

	Without feedback	With feedback	*p*‐value	Effect size
Single MVC				
Peak force in best attempt	2455 ± 510	2554 ± 478	0.042*	0.49
Mean force in best attempt	2282 ± 504	2407 ± 443	0.020*	0.62
Average peak force	2339 ± 489	2460 ± 474	0.028*	0.60
Average mean force	2148 ± 458	2313 ± 441	0.001*	0.92
Repeated MVC				
Average peak force	2118 ± 408	2284 ± 418	< 0.001*	0.85
Average mean force	1972 ± 411	2123 ± 399	< 0.001*	0.94
Peak force repetition 1	2209 ± 410	2347 ± 490	0.114	0.56
Peak force repetition 2	2196 ± 404	2365 ± 460	0.012*	0.79
Peak force repetition 3	2072 ± 397	2330 ± 463	< 0.001*	0.99
Peak force repetition 4	2082 ± 430	2251 ± 408	0.036*	0.68
Peak force repetition 5	2075 ± 2078	2191 ± 387	0.204	0.50
Peak force repetition 6	2078 ± 440	2223 ± 366	0.052	0.66
Mean force repetition 1	2076 ± 398	2223 ± 450	0.006*	0.84
Mean force repetition 2	2057 ± 421	2209 ± 417	0.030*	0.70
Mean force repetition 3	1944 ± 417	2153 ± 442	< 0.001*	0.86
Mean force repetition 4	1930 ± 433	2062 ± 414	0.096	0.58
Mean force repetition 5	1918 ± 414	2030 ± 393	0.150	0.53
Mean force repetition 6	1911 ± 445	2059 ± 363	0.036*	0.68
30 s all‐out test				
Mean force	1771 ± 347	1902 ± 325	< 0.001*	1.13
Highest 3 s window	1933 ± 402	2084 ± 388	0.030*	0.67
Lowest 3 s window	1610 ± 392	1740 ± 287	0.165	0.51
Mean force 0–5 s	1874 ± 336	2058 ± 323	< 0.001*	0.55
Mean force 5–10 s	1776 ± 290	1952 ± 301	< 0.001*	0.58
Mean force 10–15 s	1750 ± 308	1916 ± 277	< 0.001*	0.56
Mean force 15–20 s	1707 ± 328	1841 ± 248	0.010*	0.45
Mean force 20–25 s	1703 ± 316	1802 ± 254	0.120	0.34

*Note:* Average peak and mean force in the single MVC test are calculated over the three attempts. *significantly different between conditions (*p* < 0.05).

The analyses of the repeated MVCs revealed significant main effects of session for both peak (*F*(1,19) = 22.78, *p* < 0.001, and partial *η* ^2^ = 0.55) and average force (*F*(1,19) = 25.17, *p* < 0.001, and partial *η* ^2^ = 0.57). No interaction or main effect of time were found for either measure (*F*(1,19) = 0.45–1.14, *p* = 0.299–0.509, and partial *η* ^2^ = 0.02–0.06). In the repeated MVC protocol performed on the best day, the average peak force across repetitions was 8.4% higher with feedback (ES = 0.85 and *p* < 0.001) and the mean force averaged across all repetitions was 8.4% higher with feedback compared to without (ES = 0.94 and *p* < 0.001; Figure 3).

Regarding comparisons of mean force in each individual repetition, the feedback condition enhanced force output in the first three and the sixth repetition (7.1%–11.5%, ES = 0.35–0.49, and *p* = 0.006–0.036) but not in the fourth (8.2%, ES = 0.32, and *p* = 0.096) and fifth repetitions (7.0%, ES = 0.28, and *p* = 0.150). For peak force, feedback increased force output in repetitions two, three, and four (7.9%–12.9%, ES = 0.68–0.99, and *p* = 0.002–0.036) but not in repetitions one, five, and six (6.2%–8.5%, ES = 0.50–0.66, and *p* = 0.052–0.114).

The analyses of the 30 s all‐out test revealed a main effect of condition for peak force (*F*(1,19) = 19.15, *p* < 0.001, and partial *η* ^2^ = 0.50) and for the lowest (*F*(1,19) = 8.48, *p* = 0.009, and partial *η* ^2^ = 0.31) and highest 3 s time windows (*F*(1,19) = 10.34, *p* = 0.005, and partial *η* ^2^ = 0.35). No interactions or main effects of session were found (*F*(1,19) = 0.14–0.62, *p* = 0.440–0.715, and partial *η* ^2^ = 0.01–0.03). Post hoc comparisons showed that the overall mean force output increased by 8% with feedback (ES = 1.13 and *p* < 0.001). Moreover, when examining force within the highest and lowest 3 s intervals, the highest 3 s window of mean force was 8.8% higher with feedback (ES = 0.67 and *p* = 0.030), whereas the lowest 3 s mean force was not significantly different between conditions (ES = 0.51 and *p* = 0.165). Analyzing the 5 s intervals, the feedback condition consistently displayed greater force output throughout the first 20 s (9.3%–10.6%, ES = 0.45–0.58, and *p* = 0.001–0.010; Figure 4), whereas the final interval was not significantly higher (7.1%, ES = 0.34, and *p* = 0.144).

### 4.2 Reliability

The analysis of test–retest reliability between testing days indicated enhanced consistency for the condition with feedback for all outcomes collected from the single and intermittent MVC trials (Table 2). In contrast, the mean force across the 30 s all‐out test revealed no meaningful difference between the conditions (CV = 3.68% and 3.71% for the no feedback and feedback conditions, respectively), whereas the 3 s time window with the highest mean force during the all‐out test was more reliable without feedback (CV = 3.11%) than with feedback (CV = 4.08%).

**TABLE 2:** Intraclass correlation (ICC) and coefficient of variation (CV) between testing days in the same condition for single maximum voluntary contraction (MVC) attempts, the repeated MVC test, and the 30 s all‐out test.

	Without feedback		With feedback	
	ICC	CV (%)	ICC	CV (%)
Single MVC attempt				
Peak force	0.898	4.85	0.972	2.63
Mean force	0.951	3.94	0.969	2.76
Average of 3 single MVCs				
Peak force	0.955	3.58	0.973	2.57
Mean force	0.960	3.70	0.967	2.87
Average of 6 repeated MVCs				
Peak force	0.962	3.11	0.961	2.95
Mean force	0.967	3.24	0.967	2.84
30 s all‐out test				
Mean force	0.950	3.68	0.928	3.71
Highest 3 s force	0.953	3.11	0.919	4.08
Lowest 3 s force	0.841	6.92	0.891	4.30

Analyses of the intraday reliability further indicate enhanced consistency with feedback. During single MVCs, peak force ICC remained very high at 0.978, whereas CV decreased from 3.80% without feedback to 3.38% with feedback. Mean‐force ICC improved from 0.947 to 0.976, with CV dropping from 5.22% to 3.75% in the feedback condition. For the repeated MVCs, peak force ICC was 0.987 without feedback and 0.983 with feedback (CV stable at ∼4.5%) and mean‐force ICC was 0.985 versus 0.978 (CV decreased slightly from 5.30% to 5.17%). See Table 3 for the specific ICC and CV values between the three single MVC attempts and the six efforts from the repeated MVC.

**TABLE 3:** Intraclass correlation (ICC) and coefficient of variation (CV) for the single and repeated maximum voluntary contraction (MVC) with and without feedback.

	Without feedback		With feedback	
	ICC	CV (%)	ICC	CV (%)
Between 3 single MVCs				
Peak force	0.978	3.80	0.978	3.38
Mean force	0.947	5.22	0.976	3.75
Between 6 repeated MVCs				
Peak force	0.987	4.48	0.983	4.48
Mean force	0.985	5.30	0.978	5.17

*Note:* The values are calculated using data form the “best day” across three repetitions for the single MVCs and across six repetitions for the repeated MVC test.

## 5 Discussion

This study investigated the impact of real‐time visual feedback on peak and mean isometric force output and measurement reliability during IMTP testing. The results generally demonstrated that visual feedback enhances force output in single, repeated, and all‐out MVC protocols. In addition, reliability metrics assessed via CV and ICC improved in the feedback condition for most outcomes, with no meaningful changes being detected in the 30 s all‐out test. These findings support the hypothesis that real‐time visual feedback serves as an effective performance enhancer and a means of improving measurement reliability, which can further refine the IMTP protocol for both research and practical applications.

The increased force output observed across all trials with feedback aligns with existing research indicating that visual information can enhance motivation and performance in physical tasks (Concon et al. 2024; Hopper et al. 2003; Keller et al. 2014; Pacholek 2024; Wilson et al. 2017). Visual feedback likely encourages participants to aim for higher force production by facilitating real‐time adjustments during the IMTP. As Hopper et al. (2003) and Kellis and Baltzopoulos (1996) suggested, feedback provides an immediate performance reference and allows participants to more precisely adjust their exertion. In this study, the live force curve likely enabled participants to intuitively understand their performance and strive for optimal output. These findings mirror feedback effects in simpler single‐joint tasks where torque‐based feedback improved peak knee‐extension torque and EMG consistency (Hald and Bottjen 1987; Kim and Kramer 1997; Ties Molenaar et al. 2010), and in more complex, multijoint actions such as maintaining squat barbell velocity (J. J. S. Weakley et al. 2019).

Moreover, the real‐time display of force data likely heightened participants' awareness of their peak performance, motivating them to reach or even surpass previous force levels. This effect aligns with theories of motor control and motivation, which posit that real‐time feedback serves as a cognitive and motivational anchor (Keller et al. 2014; Sigrist et al. 2013; J. Weakley et al. 2023). By offering a continuous objective measure of effort, feedback may reduce the subjective uncertainty that participants can encounter in high‐intensity tasks without external reference points and allow them to consciously strive to reach a known force threshold. The feedback‐driven performance improvements may also stem from a self‐competitive effect. When participants were presented with a real‐time force curve, they may have engaged in intrinsic goal setting, where each attempt served as a benchmark to reach or even exceed in subsequent efforts. Research on goal setting supports this mechanism, demonstrating that clear measurable goals enhance physical performance by focusing attention and bolstering motivation (Williamson et al. 2022).

Focused attention may contribute to a more efficient neuromuscular activation as real‐time visual cues engage attentional resources that facilitate both motor planning and execution, potentially enhancing the recruitment of high‐threshold motor units essential for maximal force output (Shumway‐Cook and Woollacott 2001). Earlier biofeedback studies support this and suggest that visual feedback may enhance motor‐unit recruitment, decrease antagonist coactivation, and refine intermuscular coordination (Graves and James 1990; Locks et al. 2015; Lucca and Recchiuti 1983). By directing participants' focus to the force curve, the external feedback may improve the activation and sustained effort of these motor units, explaining the observed performance enhancement (Lohse et al. 2011; Wulf et al. 2001). This feedback‐enhanced neuromuscular control may be particularly advantageous in IMTP testing, where maximal force production requires continuous effort and precise motor coordination of the entire body. Without feedback, participants must rely on internal cues alone, which are likely less reliable than external quantitative information. Notably, all participants were experienced in resistance training and were expected to exert maximal effort regardless of condition, and the ICC and CV values indicated excellent reliability even in the no feedback condition, suggesting that performance was already stable and consistent. Despite this, feedback still led to greater force output, raising the question of whether participants unintentionally held back when relying on internal effort perception alone. This could suggest that even well‐trained individuals may not always reach their true maximal output without an external cue to optimize motor unit recruitment and sustain effort throughout the contraction.

Another important outcome of this study was that measurement reliability was significantly enhanced by feedback, as indicated by significant reductions in CV values across most tests. For instance, intersession test–retest reliability analyses revealed that the condition with feedback yielded consistently lower CV values across all outcomes from single and repeated MVC trials compared to the no feedback condition. A recent review of isometric strength testing recommended that reliable measures achieve an ICC above 0.8 and a CV below 10% (Brady et al. 2020). Our results met these criteria, confirming that the IMTP is a reliable test when conducted both with and without feedback. Although both conditions demonstrate acceptable reliability, the findings suggest that real‐time visual feedback helps mitigate variations arising from internal cues alone. Improved reliability is critical in strength testing, as it differentiates true performance changes from measurement error (Grgic et al. 2020; Naclerio Ayllón et al. 2009).

However, the 30 s all‐out test differed from the other test types in terms of feedback not improving overall force reliability. This may be partially explained by the test duration itself, which, despite being modeled after established all‐out anaerobic tests, such as the Wingate test (Bar‐Or 1987), remains unfamiliar to strength‐trained individuals. In contrast, a 5 s MVC may resemble the typical duration of a deadlift repetition, making it a more intuitive maximal effort task. Additionally, maintaining force output consistently over 30 s presents a greater neuromuscular challenge than shorter trials, likely increasing potential variability. Although augmented feedback can attenuate fatigue (Graves and James 1990), such benefits did not translate into improved reliability in our 30 s IMTP. This difference between short and long efforts aligns with Grover et al. (2024), who found poor reliability (ICC = 0.305) for a 60 s all‐out IMTP, whereas their shorter repeated MVC trial (ten 5 s repetitions with 10 s rest for a total work duration of 50 s) demonstrated much greater reliability (ICC = 0.790). Further supporting the reports of greater variability in longer‐duration tasks, our six‐repetition MVC test (total effort duration = 30 s) exhibited even higher ICC values (0.961–0.987). Moreover, the 30 s all‐out test in our study produced substantially higher ICC values than the 60 s test by Grover et al. (2024), reinforcing the idea that longer test durations tend to increase variability.

A potential explanation for the increased variability over extended durations in our study could be participants consciously or subconsciously using pacing strategies to maintain force rather than continually maximizing effort, which has previously been observed during repeated MVCs when participants knew the number of remaining repetitions (Halperin et al. 2014). Despite participants in our study being instructed to exert maximal effort throughout the entire trial, such pacing may have occurred. Although potentially optimizing force over time, pacing strategies could also introduce variability as participants periodically modulate exertion. These findings imply that although real‐time visual feedback generally benefits maximal output, its effects may vary with task structure and duration. These findings highlight the importance of considering test duration, participant familiarity, and sport specificity when using the IMTP to assess physical performance.

Intraday reliability further confirmed enhanced consistency with feedback as evidenced by higher ICC and reduced CV values. These reliability improvements indicate that real‐time visual feedback reduced variability in both mean and peak force output, likely by providing participants with a consistent external reference for self‐regulation during maximal efforts (Grgic et al. 2020; Weinstock‐Zlotnick et al. 2011). This external cue may allow participants to consciously or subconsciously fine tune their force output in real time, potentially reducing reliance on variable internal perceptions of effort. As a result, this could have contributed to a more stable motor execution strategy across testing sessions. Importantly, randomizing the condition order ensured that the observed differences resulted from visual feedback rather than a learning effect, reinforcing the role of external cues in improving measurement consistency.

Integrating feedback into standardized testing protocols could further refine the already well‐established IMTP protocol (Brady et al. 2020). Although some literature suggests feedback might detract from performance consistency by shifting participants' focus (Sigrist et al. 2013), this study observed the opposite effect under controlled conditions. The ability to monitor force output in real time may have provided an external reference point, helping participants maintain more stable execution rather than disrupting their effort. Importantly, the review by Sigrist et al. (2013) included papers examining a wide variety of complex and fine motor skills in addition to sporting activities, which might not all be comparable to an isometric maximal force test such as the IMTP. Our findings align more with the previous reports of visual feedback enhancing performance in sport related tasks such as jumping and weightlifting (Concon et al. 2024; Hopper et al. 2003; Keller et al. 2014; Kellis and Baltzopoulos 1996; Pacholek 2024; Weinstock‐Zlotnick et al. 2011; Wilson et al. 2017). Given the growing body of evidence supporting the benefits of visual feedback and its straightforward and cost‐effective implementation, its integration into strength assessments can be recommended as a practical tool for enhancing both performance and measurement consistency.

Despite its contributions, this study has limitations. The relatively small sample consisted solely of resistance‐trained men, which may limit generalizability. Sex differences in neuromuscular function and motivation, as well as varying levels of training experience, could modulate the effects of visual feedback. Future research should investigate whether these findings extend to women and to individuals with different strength and training backgrounds. Additionally, the current study focused specifically on real‐time feedback during isometric testing in a deadlift‐like position, assessing its effects on reliability and performance. Future research should examine whether similar feedback approaches are feasible and effective in other exercises, contraction types, or delivery methods, including feedback provided after completing an attempt. Finally, this study did not investigate underlying neuromuscular mechanisms, such as motor unit firing frequency or muscle activation patterns. Although this was beyond this study's scope, previous biofeedback studies offer insight into how visual feedback may reshape neuromuscular control. For example, Locks et al. (2015) found that force‐based feedback produced greater vastus lateralis electromyographic (EMG) adaptations than EMG feedback, suggesting enhanced agonist drive. Graves and James (1990) showed that augmented feedback increased peak force and reduced fatigue in novel digit tasks, which imply refined motor‐unit recruitment. Lucca and Recchiuti (1983) demonstrated that EMG‐feedback training yielded larger torque gains than exercise alone. Collectively, these findings suggest that visual feedback during isometric tasks might boost high‐threshold motor‐unit activation, reduce antagonist coactivation, and alter intermuscular coordination. Future IMTP studies should probe these mechanisms with integrated force–EMG recordings.

## 6 Conclusion

In conclusion, real‐time visual feedback improved both force output and measurement reliability during IMTP testing, emphasizing its potential for enhancing performance assessments. These findings support the inclusion of visual feedback in isometric strength testing to provide a more accurate representation of maximal force capabilities. Future research should investigate how different feedback modalities and implementation strategies influence training adaptations and performance outcomes across various strength assessment protocols.

## Ethics Statement

The protocols conformed to the latest revision of the Helsinki declaration, followed Norwegian and local university laws and regulations, and were processed by the Norwegian Center for Research Data (SIKT; reference: 140165).

## Conflicts of Interest

The authors declare no conflicts of interest.

## Data Availability

All data related to this work are attainable from the corresponding author upon reasonable request.
